# Retraction: Tumor growth suppression by inhibiting both autophagy and STAT3 signaling in HNSCC

**DOI:** 10.18632/oncotarget.28865

**Published:** 2026-04-24

**Authors:** Teng-Fei Fan, Lin-Lin Bu, Wei-Ming Wang, Si-Rui Ma, Jian-Feng Liu, Wei-Wei Deng, Liang Mao, Guang-Tao Yu, Cong-Fa Huang, Bing Liu, Wen-Feng Zhang, Zhi-Jun Sun

**Affiliations:** ^1^The State Key Laboratory Breeding Base of Basic Science of Stomatology and Key Laboratory of Oral Biomedicine Ministry of Education, Wuhan University, Wuhan, China; ^2^Department of Oral and Maxillofacial-Head and Neck Oncology, School and Hospital of Stomatology, Wuhan University, Wuhan, China; ^*^These authors have contributed equally to this work

**This article has been retracted:** Oncotarget has completed its investigation of this paper, which identified several instances of internal image duplication, overlap, and the reuse of images in a later published article by the same group [1], despite representing different experiments:

- Figure 1A: The fluorescence microscopy image (Hoechst 33258 staining) is a duplicate of the image in Supplementary Figure 2A.
- Figure 1D: The Western blot for STAT3 is a duplicate of the STAT blot in Supplementary Figure 2C. This image was also reused in Figure 4B of a 2016 paper by the same group [1].
- Figure 2B: The Western blot for Beclin1 is a duplicate of the pAkt blot in Figure 4A.
- Figure 2D: The Western blots for GAPDH and LC3 are duplicates of the images in Figures 3A, 3C and 3E, respectively.
- Figure 3C: The Western blot for LC3 overlaps with the image in Figure 3E.
- Figure 3E: The Western blot for GAPDH is a duplicate of the image in Supplementary Figure 1B.
- Figure 4A, 4C: The Western blots for GAPDH and Erk1/2 are duplicates of the corresponding images in Figure 4D.
- Figure 5C: The third and fourth IHC images for the p62 stained group overlap; additionally, the second image overlaps with the IHC image for LAMP2 in Figure 6A.
- Supplementary Figure 2A: The Tunnel assay control image (0 uM) overlaps with the 50 uM image.

The authors initially requested a correction; however, given the volume of inconsistencies, they subsequently requested a retraction. The editorial decision has been made to retract the article, and the authors have been notified of this final decision, but have not responded.

Original article: Oncotarget. 2015; 6:43581–43593. 43581-43593. https://doi.org/10.18632/oncotarget.6294
